# Sex differences in total brain volume in a cognitively unimpaired elderly population

**DOI:** 10.1192/j.eurpsy.2021.1091

**Published:** 2021-08-13

**Authors:** M. Buchpiguel, G. Busatto, P. Rosa, P. Squarzoni, F. Duran, J. Tamashiro-Duran, C. Leite, P. Lotufo, M. Scazufca, T. Alves

**Affiliations:** 1 Faculty Of Medical Sciences Of São Paulo, Santa Casa de São Paulo School of Medical Sciences, São Paulo, Brazil; 2 Lim21 Laboratory Of Psychiatric Neuroimaging (lim-21), Department And Institute Of Psychiatry, Faculty of Medicine, University of São Paulo, São Paulo, Brazil; 3 Department & Institute Of Psychiatry, Faculty of Medicine, University of São Paulo, São Paulo, Brazil; 4 Department Of Radiology, Faculty of Medicine, University of São Paulo, São Paulo, Brazil; 5 Clinical Research And Epidemiology Unity, Faculty of Medicine, University Hospital, University of São Paulo, São Paulo, Brazil

**Keywords:** Magnetic Resonance Imaging, Grey Matter, White Matter, Sexual Dimorphism

## Abstract

**Introduction:**

Although a large number of studies have shown brain volumetric differences between men and women, only a few investigations to date have analyzed brain tissue volumes in representative samples of the general elderly population.

**Objectives:**

We investigated differences in gray matter (GM), white matter (WM) and intracranial volumes (ICVs) between sexes in individuals above 66 years old using structural magnetic resonance imaging (MRI).

**Methods:**

Using FreeSurfer version 5.3, we automatically obtained the ICVs, GM and WM volumes from MRI datasets of 84 men and 92 women. To correct for interindividual variations in ICV, GM and WM volumes were adjusted with a method using the residuals of a least-square-derived linear regression between raw volumes and ICVs. We then performed an ANCOVA comparing men and woman including age and years of schooling as confounding factors.

**Results:**

Women had a lower socioeconomic status overall and fewer years of schooling than men. The comparison of unadjusted brain volumes showed larger GM and WM volumes in men. After the ICV correction, the adjusted volumes of GM and WM were larger in women.

**Conclusions:**

After the ICV correction and taking into account differences in socioeconomic status and years of schooling, our results confirm previous findings of proportionally larger GM in women, as well as larger WM volumes. These results in an elderly population indicate that brain volumetric differences between sexes persist throughout the aging process. Additional studies combining MRI and other biomarkers are warranted to identify the hormonal and molecular bases influencing such differences.

